# Risk Factors for Sepsis and Pulmonary Embolism Mortality in Burn Patients by Using a Competing Risk Model: A Machine Learning Approach

**DOI:** 10.1155/bmri/7224574

**Published:** 2026-08-12

**Authors:** Fatemeh Javanmardi, Zahra Shayan, Amir Emami, Soheila Khodakarim

**Affiliations:** ^1^ Department of Biostatistics, School of Medicine, Shiraz University of Medical Sciences, Shiraz, Iran, sums.ac.ir; ^2^ Burn and Wound Healing Research Center, Microbiology Department, Shiraz University of Medical Sciences, Shiraz, Iran, sums.ac.ir

**Keywords:** burn, competing risk, penalized methods, pulmonary embolism, sepsis

## Abstract

**Introduction:**

Severe burn injury remains a prevalent cause of morbidity and mortality over the last several decades. Various potential complications significantly contribute to mortality in burn patients. Sepsis and pulmonary embolism (PE) are among the leading causes of death following burn injury.

**Methods:**

This retrospective study was conducted at a burn center in southwest Iran. In the current study, competing risk analysis was performed. Two events were considered death due to PE, which is the event of interest, and the competing event defined as death due to sepsis. Discharge was considered as a censor. Hazard ratios (HR) and standard errors (SE) were used to estimate the risk of death.

**Results:**

The study included 623 patients, with a total of 458 deaths (73.5%). Among them, 17 (2.7%) were due to PE, and 441 (70.8%) were attributed to sepsis‐related mortality. Lower limb burns were the most frequent injury site among survivors (77.0%) and PE cases (82.3%). Flame burns were the most common etiology in all groups. The need for mechanical ventilation is also associated within increasing hazard of mortality in both events. Patients with burns in the lower limbs are more likely to die from PE (HR = 3.15) and sepsis (HR = 2.10). A higher RBC count was associated with a decreased risk of mortality from both PE (HR: 0.66) and sepsis (HR: 0.53). Similarly, hemoglobin levels had a modest protective effect against both causes of death.

**Conclusion:**

These results highlight the complex relationship between burn characteristics, laboratory indicators, and demographic factors in determining mortality risk. Both clinical and laboratory parameters, as well as burn site and severity, play important roles in predicting outcomes in burn patients.

## 1. Introduction

Severe burn injury remains a significant cause of morbidity and mortality worldwide over recent decades. Although the overall incidence of burn injuries has decreased, no substantial change has been observed in the relative mortality rate. Annually, more than 11 million people are affected by burns, making them the fourth most common type of trauma globally [1, 2]. Following burn injury, a profound systemic inflammatory response syndrome (SIRS) provokes nearly every organ in the body. Approximately 5% of mortality due to severe burns is associated with immune compromise, infection, sepsis, multiorgan failure, hyperinflammation, and coagulopathy [3]. Additionally, secondary injury progression following burn shock further exacerbates tissue damage in these patients [4].

Changes in coagulation pathways can lead to microvascular clotting and potentially worsen patients′ outcomes. This complication can arise from pulmonary embolism (PE), which is a life‐threatening condition induced by burn‐related factors [5]. The incidence rate of PE after burn injuries is estimated to be 0.05%–1.4%. According to the American Burn Association′s National Burn Repository, the overall incidence of PE is approximately 0.18%, which increases to 0.38% in patients with a total body surface area (TBSA) burned greater than 10% [6].

On the other hand, disruption of the skin barrier places patients at a high risk of infection and significantly increases mortality due to sepsis. In fact, infection is the leading cause of death beyond 72 h after burn injury [7]. The incidence of sepsis is varied from 3% to 30% for patients with TBSA more than 20%. Burn patients exhibit a higher incidence of sepsis compared with other trauma. These vulnerabilities stem from the initial damage, systemic responses, and prolonged recovery process [8].

Various complications contribute significantly to mortality in burn patients. Sepsis and PE are among the leading causes of death following burn injury. Applying appropriate statistical methods to model outcomes in these patients, who experience complex and interrelated conditions, is inherently challenging [9]. In survival analysis, multiple events may occur, with one event potentially preventing or changing the probability of others. Standard statistical methods, such as Cox regression, are not proper methods to handle this complexity. Competing risks models are specifically designed to address this issue by estimating the probability of a specific event while accounting for the presence of competing events [10].

The application of competing risks analysis represents a significant advancement in understanding the complex dynamics of recovery and mortality in patients experiencing multiple outcomes [11]. However, careful consideration is required regarding model complexity, particularly when one of the outcomes is rare. Penalized methods, as a class of machine learning techniques, can enhance both the interpretability and predictive performance of statistical models. These approaches are especially valuable in the context of rare events, which occur less frequently than other outcomes [12, 13]. The present study is designed to address this challenge by providing reliable predictions in burn patients who may experience multiple competing events, including death due to PE (a rare event), death due to sepsis, or discharge. The aim of this study is to identify predictors associated with each outcome by applying penalized variable selection methods within a competing risks framework, with particular emphasis on death due to PE as a rare event.

## 2. Methods

This retrospective study was conducted in the center of burn in southwest of Iran affiliated with Shiraz University of Medical sciences. After approval by the ethical committee of the University (Ethical code: IR.SUMS.REC.1402.265), the data of 623 burn patients who were admitted during 2020 were collected.

The inclusion criteria were defined to ensure that patients had sufficient exposure to hospitalization and were at risk for the outcomes of interest. These criteria included early admission postburn, defined as admission within 3 days of the burn injury, in order to capture early systemic responses and minimize bias related to delayed hospital care; adequate hospital stay, defined as a length of stay (LOS) greater than 48 h, to ensure sufficient observation time for the development of sepsis, PE, or discharge; and a confirmed burn diagnosis, defined as documented burn injuries affecting anybody region. The exclusion criteria were applied to improve the accuracy of outcome attribution and ensure data completeness. These included deaths unrelated to the study outcomes, whereby patients whose cause of death was not attributable to sepsis or PE were excluded to focus the analysis on these competing risks; incomplete or missing baseline data, including laboratory, clinical, or demographic information at admission, which were excluded to preserve the validity of the statistical modeling; and extremely short hospital stay (< 48 h), as patients discharged or deceased shortly after admission may not have had sufficient time to develop the outcomes of interest. By applying these criteria, we ensured a homogeneous cohort in which the timing, severity, and outcomes of burn‐related complications could be reliably analyzed. These criteria also allowed for accurate estimation of the incidence and predictors of mortality due to sepsis and PE, while minimizing confounding factors associated with unrelated deaths or incomplete data.

Based on the definition provided by centers for disease control and prevention (CDC), sepsis was defined as changing in burn wound appearance including rapid scar separation or discoloration to dark brown; black or purple, accompanied with histological confirmation of microorganism invasion into nearby healthy tissue or a positive blood culture. Clinical signs and symptoms regarding sepsis include fever (temperature more than 38^°^C); hypertension (systolic pressure less than 90 mmHg), or oliguria (less than 20 cm^3^/h). Computed tomography (CT) pulmonary angiography was used for the diagnosis of PE, providing an assessment of the location and degree of clot burden [14, 15].

### 2.1. Variables

In the present study, a competing risks analysis framework was applied. Two events were considered: death due to PE as the event of interest, and death due to sepsis as the competing event. Discharge was treated as a censoring event. The time‐to‐event variable was defined as the duration from hospital admission to the occurrence of death (due to PE or sepsis) or discharge. The data set contains information about age, sex, final status (discharge, death due to PE, death due to sepsis), LOS, TBSA, the site of burn (upper limbs, lower limbs, torso, head, and neck), the type of burn (scald, chemical, flame, explosion, and electrical), using mechanical ventilator, and laboratory factors including albumin, blood urea nitrogen (BUN), creatinine, white blood cell (WBC), red blood cell (RBC), hemoglobin (HB), sodium (Na), and potassium (K).

### 2.2. Statistical Analysis

Four penalized methods including Least Absolute Shrinkage and Selection Operator (LASSO), smoothly clipped absolute deviations (SCAD), minimax concave penalty (MCP), and adaptive LASSO were applied to detect the important and significant predictors of two events. Model performance was evaluated using the Bayesian information criterion (BIC). The model with the lowest BIC and best performance was LASSO for choosing the predictive factors associated with death‐related PE and MCP for choosing the predictive factors associated with death‐related sepsis. Therefore, only findings related to LASSO and MCP are shown in the results section and the findings related to other methods are provided in the Supporting Information. Quantity variables presented as mean ± standard deviation and qualitative ones presented as frequency (percentage). LOS reported as median (interquartile range). Hazard ratio (HR) and standard error (SE) were used to report the risk of death. All the statistical analyses were performed by R software, and packages crrp and fastcmprsk.

## 3. Results

The study included 623 burn patients, of whom 458 (73.5%) died, including 17 (2.7%) due to PE and 441 (70.8%) due to sepsis. The mean age was highest in the PE group (37.93 ± 16.96 years), followed by the sepsis group (31.29 ± 15.24 years), and the discharge group (27.28 ± 19.49 years). Lower limb burns were the most frequent injury site among survivors (77.0%) and PE cases (82.3%), whereas torso burns (53.7%) and head and neck burns (33.3%) were more frequent among patients who died due to sepsis. Flame burns were the most common etiology in all groups. Regarding laboratory findings, HB and RBC levels were lower in deceased patients compared with survivors. Creatinine levels were highest in PE patients (1.19 ± 0.56 mg/dL), followed by sepsis patients (1.04 ± 0.46 mg/dL) and survivors (0.93 ± 0.32 mg/dL). WBC levels were higher in the PE group (13.51 ± 7.98 × 10^3^/*μ*L) compared with the sepsis and discharge groups. TBSA was substantially higher in patients who developed PE. Mechanical ventilation was more frequently required among nonsurvivors. Flame burns were the predominant burn type across all outcome groups. Table 1 summarizes baseline demographic, clinical, and laboratory characteristics.

**Table 1:** Demographic, clinical, and laboratory factors in burn patients.

Variables	Discharge (*n* = 165)	Death due to pulmonary embolism (*n* = 17)	Death due to sepsis (*n* = 441)
Demographic variables			
Age (years)	27.28 ± 19.49	37.93 ± 16.96	31.29 ± 15.24
Sex (male: female)	82:83 (49.69%: 50.30%)	12:5 (70.58%: 29.41%)	282:159 (63.94%: 36.05%)
Clinical variables			
Length of hospital stay (day)	9 (8)	6 (2)	12 (11)
Mechanical ventilation	36 (21.81%)	9 (52.94%)	163 (36.96%)
TBSA (%)	35.29 ± 20.61	80.41 ± 16.74	68.76 ± 20.28
Site burn			
Upper limbs	3 (1.8%)	1 (5.88%)	18 (4.08%)
Lower limb	127 (77.0%)	14 (82.3%)	39 (8.8%)
Torso	22 (13.33%)	1 (5.88%)	237 (53.74%)
Head and neck	13 (7.87%)	1 (5.88%)	147 (33.33%)
Etiologies of burn			
Scald	43 (26.06%)	3 (17.64%)	108 (24.48%)
Chemical	11 (6.66%)	1 (5.88%)	28 (6.34%)
Flame	62 (37.57%)	6 (35.29%)	204 (46.25%)
Explosion	34 (20.60%)	5 (29.41%)	93 (21.08%)
Electrical	15 (9.09%)	2 (11.76%)	8 (1.81%)
Laboratory variables			
WBC (10^3^/*μ*L) ^∗^	11.35 ± 4.20	13.51 ± 7.97	11.48 ± 4.97
RBC (million/*μ*L)	5.28 ± 1.45	3.91 ± 0.98	4.16 ± 0.95
HB (g/dL)	13.41 ± 4.37	10.95 ± 2.23	11.65 ± 3.32
BUN (mg/dL)	16.01 ± 8.56	21.91 ± 8.09	18.57 ± 10.39
Creatinine (mg/dL)	0.93 ± 0.32	1.19 ± 0.55	1.04 ± 0.46
NA (mmol/L)	138.14 ± 32.38	134.74 ± 26.82	137.65 ± 44.03
K (mmol/L)	4.07 ± 0.6	4.54 ± 1.35	4.12 ± 0.71
Albumin (g/dL)	4.69 ± 0.76	4.09 ± 1.04	4.10 ± 0.98

*Note:* Data are presented as mean ± standard deviation and frequency (%), and time is reported as median (interquartile range). LOS is reported as median (IQR). Death due to pulmonary embolism is the interested event; death due to sepsis is the competing event.

Abbreviation: TBSA, total burn surface area.

Table 2 presents the HR and SE for factors associated with mortality due to PE and sepsis in burn patients, selected through the LASSO and MCP variable selection methods, respectively. Several demographic, clinical, and laboratory factors influence the risk of mortality in these patients.

**Table 2:** Risk of death due to pulmonary embolism and sepsis in burn patients, based on variable selection methods in competing risk analysis.

	Death due to pulmonary embolism^a^		Death due to sepsis^b^	
	Hazard ratio	Standard error	Hazard ratio	Standard error
Age (years)	1.01	0.011	0.99	0.0028
Sex (male)	1.85	0.21	1.44	0.15
Female	**Reference**			
TBSA (%)	1.18	0.009	1.98	0.0027
Mechanical ventilation	1.68	0.01	1.27	0.12
Site burn				
Upper limb	**Reference**			
Lower limb	3.15	0.01	2.10	0.29
Torso	—	—	0.086	0.26
Head and neck	2.01	0.01	—	—
Cause burn				
Scald	**Reference**			
Chemical	1.06	0.02	1.00	0.51
Flame	1.31	0.21	2.20	0.12
Explosion	1.26	0.05	3.19	0.14
Electrical	1.02	0.004	0.75	0.23
Laboratory factors				
WBC (×*μ*L/10^3^)	—	—	—	—
RBC (million/*μ*L)	0.66	0.16	0.53	0.063
HB (g/dL)	0.96	0.052	0.97	0.019
BUN (mg/dL)	—	—	1.00	0.0058
Creatinine (mg/dL)	—	—	0.89	0.001
NA (mmol/L)	0.99	0.001	0.99	0.0011
K (mmol/L)	1.07	0.12	1.01	0.082
Albumin (g/dL)	0.69	0.25	0.93	0.15

*Note:* The symbol (—) indicates not selected as important factor. Death due to pulmonary embolism is the event of interest; death due to sepsis is the competing event.

^a^Based on the LASSO variable selection method.

^b^Based on the MCP variable selection method.

Age showed only a marginal association with mortality, with a slight increase in PE‐related mortality (HR = 1.01) and a slight decrease in sepsis‐related mortality (HR = 0.99). Male sex was associated with higher mortality risk for both PE (HR = 1.85) and sepsis (HR = 1.44) compared with females. TBSA was another selected predictor of mortality, with a stronger effect for sepsis‐related deaths (HR = 1.98) than for PE‐related deaths (HR = 1.18). The need for mechanical ventilation was also associated with increased hazard of death in both events (HR = 1.68 for PE; HR = 1.27 for sepsis).

Burn location was an important predictor of mortality. Lower limb burns were associated with higher risk of death from PE (HR = 3.15) and sepsis (HR = 2.10) compared with upper limb burns. Head and neck burns increased the risk of PE‐related mortality (HR = 2.01), whereas torso burns were associated with a substantially lower risk of sepsis‐related mortality (HR = 0.086), effectively reducing mortality risk by 91%. Burn etiology also plays a crucial role in mortality risk. Compared with scald burns, flame burns increase the risk of mortality in both PE (HR = 1.31) and sepsis (HR = 2.20). Explosions are particularly associated with a high risk of sepsis‐related mortality (HR = 3.19), whereas their impact on PE‐related mortality is more moderate (HR = 1.26). Electrical burns were associated with only a 2% increase in PE‐related mortality risk. It may be associated with a lower risk of sepsis‐related mortality (HR = 0.75). A higher RBC count was associated with a decreased risk of mortality from both PE (HR: 0.66) and sepsis (HR: 0.53). Similarly, HB levels had a modest protective effect against both causes of death (HR: 0.96 for PE and 0.97 for sepsis), although the HR was small. BUN and creatinine level are important markers of kidney function and metabolic stress. BUN was only reported for sepsis‐related mortality and did not appear to influence risk (HR: 1.00). Creatinine was not identified as a predictor for PE‐related mortality but exhibited a modest protective effect for sepsis‐related mortality (HR = 0.89). Electrolytes were also evaluated for their association with mortality risk. Na levels showed negligible effects on mortality due to either PE or sepsis (HR = 0.99 for both), indicating that minor fluctuations in Na concentrations do not substantially affect outcomes in burn patients. K showed a small increase in risk for PE‐related mortality (HR = 1.07) and minimal effect on sepsis mortality (HR = 1.01). White blood cell count was not selected as a predictor for mortality. Finally, lower serum albumin levels were associated with increased mortality risk in both PE (HR = 0.69) and sepsis (HR = 0.93), highlighting its potential role as a prognostic marker in burn patients.

## 4. Discussion

This retrospective study examined the two primary causes of death following burn injuries—sepsis and PE—using a competing risks framework. The incidence of PE, a rare event in this cohort, was estimated at 2.7%. To address the challenge of rare outcomes, penalized variable selection methods were employed, allowing identification of the most important covariates for each event.

The overall mortality rate observed in this cohort (73.5%) appears higher than that reported in many burn studies and should be interpreted in the context of the study setting and patient population. This study was conducted in a specialized tertiary referral burn center that receives a substantial proportion of severe and complicated burn cases from a wide geographic region. In addition, many patients had extensive burn injuries, as reflected by the high TBSA values among nonsurvivors, and a considerable proportion required mechanical ventilation, indicating critical illness. Therefore, the observed mortality rate likely reflects the severity of the underlying patient population rather than the general burn population, and caution should be exercised when comparing these findings with studies conducted in less severely injured cohorts.

In the current study, RBC, HB, Na, K, albumin, sex, TBSA, age, burn site, mechanical ventilation, and burn type were identified as selected predictors of mortality due to PE. For sepsis‐related mortality, RBC, sex, and burn site were the most influential predictors, whereas other covariates demonstrated weaker associations. The higher mortality observed in septic patients compared with noninfected individuals is consistent with prior literature [16, 17]. Disruption of the skin barrier—the body′s first line of defense—creates an opportunistic environment for pathogens, increasing susceptibility to sepsis and making other organs more vulnerable to secondary insults. These findings underscore the importance of early risk assessment and appropriate venous thromboembolism (VTE) prophylaxis in burn patients to improve clinical outcomes [18].

Extensive burns expose the body to a profound stress response, including hypermetabolism and immunosuppression, making patients more susceptible to complications. Early diagnosis and appropriate treatment are critical to improving survival outcomes. In the current study, burn site emerged as an important predictor of mortality due to PE, with the lower limbs being the most frequently affected site. This finding may reflect the impact of burn location on mobility, circulation, and susceptibility to infection. Consistent with this, previous studies have reported a significantly increased relative risk of VTE in patients with burns affecting the lower limbs, eyes, and trunk, likely due to immobilization contributing to higher VTE rates [19, 20]. In addition, inflammatory responses following burn injury can create a hypercoagulable state, further promoting PE. Thermal injury also causes vascular damage, which directly contributes to thrombus formation [21]. The types of burn were confirmed as a predictive factor for both cause of death in this study. Flame was the most prevalent type of burn in patients who experienced the PE. This may be likely due to deeper tissue damage and prolonged exposure to high temperatures. Previous research has also demonstrated a strong association between flame burns and sepsis [22]. Electrical burns have been identified as an independent risk factor for PE in other studies, potentially due to differences in tissue damage and susceptibility to thrombosis compared with other burn types [23]. This could be due to differences in tissue damage and infection susceptibility compared with other burn types. In another study, it was indicated contact with chemical substance led to tissue necrosis and toxicity, and so tissue damage is more associated with increasing the risk of infection and sepsis [24]. In the present study, patients who died were generally older than those who survived, consistent with prior findings. The risk of PE increases with age, as older patients are more susceptible to deep vein thrombosis due to prolonged immobility and preexisting comorbidities. Kangarloo et al. reported that each additional year of age increases the risk of mortality, particularly in patients with TBSA burns exceeding 20% [25].

Generally, sex differences play an important role in the epidemiology, etiology, treatment, and outcome of burn injuries [26]. Optimizing treatment requires a comprehensive understanding of these differences. In the present study, sex was identified as an important predictor, suggesting that it may influence the underlying physiological response to severe burns and related complications.

TBSA is another critical metric, which was identified as a predictor for both evaluated outcomes in this study. As it was presented higher, TBSA was associated with increased risk of mortality. Other studies found TBSA as a pivotal determinant that increases the risk of death and complications like sepsis and VTE, exacerbating the mortality rate [27, 28]. Pavoni et al. reported the incidence of PE at 4%, and they found that higher TBSA increases the respiratory complications and PE [29]. Early wound excision and grafting have been suggested as effective interventions to reduce burn‐related complications and improve outcomes. These findings underscore the well‐established link between extensive burn injuries and systemic inflammatory responses, which predispose patients to severe infections and sepsis. Mechanical ventilation was also identified as a major risk factor for PE in burn patients, highlighting the impact of respiratory complications in critically ill patients. This observation is supported by previous research; in the study by Bordeanu‐Diaconescu et al., approximately half of VTE cases occurred in mechanically ventilated patients [6]. In another study, it was reported that mechanical ventilation increases the risk of VTE two times more [30]. The increased risk is likely related to prolonged immobilization and the severity of illness in these patients. According to current results, albumin has a critical role in the mortality rate due to sepsis and PE. This finding aligns with other studies. Albumin deficiency is associated with impaired immunity, which causes susceptibility to infection and further inflammation. Studies indicate burn patients with albumin levels below 2 g/dL are 80% more at risk of mortality, which indicates the importance of hypoalbuminemia in this group of patients. Burns create a systemic inflammation for these patients, this condition enhances the coagulation pathway and so thromboembolic events are most probable to occur. Lower albumin levels have been widely recognized as markers of malnutrition and systemic inflammation, both of which are associated with worse clinical outcomes in critically ill burn patients [31–33]. Although there is no direct study regarding albumin level in burn patients with PE, the potential association may be helpful in this context.

Our results also identified creatinine as a protective factor against sepsis‐related mortality. Lower creatinine levels, which may reflect better renal function, appear to be associated with a reduced risk of sepsis‐related death. Rehou et al. declared rising creatinine level in burn patients increase the risk of sepsis and mortality three times. They suggest increasing creatinine is a warning sign of kidney failure and multiorgan dysfunction in burn victims [34].

In our results, RBC retained as an important predictor with two causes of death. Other studies reported similar results, which is decreasing RBC is strongly correlated with increasing mortality rate in burn patients; it reflects the severity of systemic inflammation [35, 36]. This suggests that anemia or reduced erythropoiesis might contribute to worse outcomes in burn patients. Surgical interventions, although necessary for burn care, can exacerbate systemic stress and inflammatory responses. Burn injuries also increase blood viscosity due to fluid loss, promoting venous stasis and clot formation, which may further reduce RBC counts through hemolysis and bone marrow suppression [37]. Additionally, increased coagulation cascade activity and platelet activation indicate a systemic prothrombotic state caused by burn injury. This condition is further worsened by RBC alterations, increasing the risk of PE. Ultrarestrictive RBC transfusion strategies (HB threshold < 7 g/dL) may be acceptable in extensive burn patients, but thresholds below 6 g/dL are associated with significantly higher hospital mortality in septic patients [38]. Generally, patients who die following severe burn injury exhibit lower HGB, HCT, and RBC counts compared with survivors during the first week postinjury. Red cell distribution width (RDW) has also been associated with mortality in septic burn patients [39].

Although many studies have evaluated the risk factors of death in burn patients, but a proper statistical analysis set apart this study from previous ones. Similar findings between studies add extra impotence to identifying the risk factors in burn patients who had complex condition. The consistency of our findings with previous research further underscores the importance of these factors in a population with highly complex clinical conditions. The main limitation of this study is its focus on selected laboratory parameters, without considering other potentially important factors such as body mass index (BMI), comorbidities (e.g., diabetes mellitus, hypertension, cancer, and asthma), smoking status, and socioeconomic factors. For future studies, it is suggested to use sepsis‐specific biomarkers and clinical scores, which could allow for more precise risk stratification and improve the predictive accuracy of competing risk models in burn patients.

## 5. Conclusion

Taken together, these results demonstrate the complex relationship between mortality risk and burn patients, with laboratory indicators, burn features, and demographic variables all having an impact on results. Compared with PE, TBSA showed a stronger association with sepsis‐related mortality, highlighting the critical need for infection control and management of systemic inflammation during the postburn phase. In addition, the importance of mechanical ventilation and burn site emphasizes the necessity of mobility measures and optimal respiratory support to reduce risk. Furthermore, the differential impact of burn etiology suggests that individualized treatment plans are necessary to address the specific challenges posed by different types of burns. Future research should explore the mechanisms underlying these associations and develop targeted interventions to reduce mortality risk in this vulnerable population.

## Author Contributions

F.J. and Z.S. were responsible for the design, analysis, and interpretation. Z.S. supervised the study.

## Funding

No funding was received for this manuscript.

## Disclosure

The authors take full responsibility for the content, interpretations, and conclusions presented in this manuscript. All authors read and approved the final manuscript.

## Ethics Statement

This study was conducted according to the principles expressed at Shiraz University of Medical Sciences, and it was approved by the local Ethics Committee of Shiraz University of Medical Sciences by the code IR.SUMS.REC.1402.265. Consent to participate is not applicable for this simulation study.

## Conflicts of Interest

The authors declare no conflicts of interest.

## Supporting information


**Supporting Information** Additional supporting information can be found online in the Supporting Information section. This file contains two supplementary tables: Table S1 presents the risk of death due to pulmonary embolism based on four penalized methods, and Table S2 presents the risk of death due to sepsis based on four penalized methods.

## Data Availability

The data that support the findings of this study are available from the corresponding author upon reasonable request.
