# Supplementary material for: Risk Factors for Sepsis and Pulmonary Embolism Mortality in Burn Patients by Using a Competing Risk Model: A Machine Learning Approach
Source: Biomed Res Int. 2026 Aug 12;2026:7224574. doi: 10.1155/bmri/7224574 (PMC13464429; doi:10.1155/bmri/7224574)
Supplement: Supplementary file 1 [file BMRI-2026-7224574-s001.docx]

## TableS1. Risk of death due to pulmonary embolism based on four penalized methods

|  | LASSO | | SCAD | | MCP | | Adaptive LASSO | |
| --- | --- | --- | --- | --- | --- | --- | --- | --- |
|  | HR | SE | HR | SE | HR | SE | HR | SE |
| Age | 1.01 | 0.011 | 1.01 | 0.013 | - | - | 1.05 | 0.03 |
| Sex (Male) | 1.85 | 0.21 | 1.5 | 0.59 | 1.49 | 0.59 | 0.93 | 0.12 |
| Female | **Reference** | | | | | | | |
| TBSA | 1.18 | 0.009 | 1.35 | 0.095 | 1.12 | 0.23 | 1.55 | 0.044 |
| Mechanical ventilation | 1.68 | 0.01 | 1.73 | 0.012 | 2.10 | 0.11 | - | - |
| Site Burn |  | | | | | | | |
| Upper | **Reference** | | | | | | | |
| Lower | 3.15 | 0.01 | 3.24 | 0.33 | 4.71 | 0.18 | - | - |
| Torso | - | - | - | - | - | - | - | - |
| Head and Neck | 2.01 | 0.01 | 2.12 | 0.21 | - | - | 1.99 | 0.086 |
| Cause Burn |  | | | | | | | |
| Scald | **Reference** | | | | | | | |
| Chemichal | 1.06 | 0.02 | 1.01 | 0.2 | 1.11 | 0.1 | - | - |
| Flame | 1.31 | 0.21 | 1.25 | 0.17 | 1.4 | 0.23 | 1.39 | 0.041 |
| Explosion | 1.26 | 0.05 | 1.36 | 0.21 | - | - | - | - |
| Electrical | 1.02 | 0.004 | 1.01 | 0.19 | - | - | - | - |
| WBC (×µL/10³) | - | - |  |  | 0.96 | 0.051 | - | - |
| RBC (million/µL) | 0.66 | 0.16 | 1.58 | 0.052 | 1.58 | 0.19 | 1.10 | 0.064 |
| HB(million/µL) | 0.96 | 0.052 | 1.07 | 0.185 | 1.09 | 0.099 | - | - |
| BUN (million/µL) | - | - |  |  | 1.02 | 0.03 | - | - |
| Creatinine (mg/dL) | - | - |  |  | 0.7 | 0.74 | - | - |
| NA(mmol/L) | 0.99 | 0.001 | - | - | - | - |  | - |
| K(mmol/L) | 1.07 | 0.12 | - | - | 1.23 | 0.508 | 1.23 | 0.083 |
| Albumin(g/dL) | 0.69 | 0.25 | 0.73 | 0.27 | 0.86 | 0.27 | 0.71 | 0.12 |

## TableS2. Risk of death due to sepsis based on four penalized methods

|  | LASSO | | SCAD | | MCP | | Adaptive LASSO | |
| --- | --- | --- | --- | --- | --- | --- | --- | --- |
|  | HR | SE | HR | SE | HR | SE | HR | SE |
| Age | 1.00 | 0.009 | 1.00 | 0.01 | 0.99 | 0.002 | 0.99 | 0.02 |
| Sex (Male) | 1.44 | 0.13 | 1.43 | 0.13 | 1.44 | 0.15 | 1.96 | 0.12 |
| Female | **Reference** | | | | | | | |
| TBSA | 1.02 | 0.0026 | 1.02 | 0.002 | 1.98 | 0.0027 | - | - |
| Mechanical ventilation | 1.4 | 0.1 | 1.32 | 0.02 | 1.27 | 0.12 | - | - |
| Site Burn |  | | | | | | | |
| Upper | **Reference** | | | | | | | |
| Lower | 1.53 | 0.098 | 2.12 | 0.01 | 2.10 | 0.29 | 2.3 | 0.01 |
| Torso | - | - | - | - | 1.2 | 0.26 | - | - |
| Head and Neck | 1.12 | 0.01 | 1.5 | 0.12 | - | - | 1.4 | 0.02 |
| Cause Burn |  | | | | | | | |
| Scald | **Reference** | | | | | | | |
| Chemichal | 1.3 | 0.01 | 1.6 | 0.04 | 1.00 | 0.51 | - | - |
| Flame | 1.5 | 0.08 | 1.12 | 0.01 | 2.20 | 0.12 | - | - |
| Explosion | 1.42 | 1.02 | 1.63 | 0.08 | 3.19 | 0.14 | - | - |
| Electrical | - | -- |  | - | 0.75 | 0.23 | - | - |
| WBC (×µL/10³) | 1.00 | 0.09 | 1.10 | 0.01 | 0.007 | 0.01 | - | - |
| RBC (million/µL) | 0.94 | 0.05 | 0.93 | 0.062 | 0.53 | 0.063 | 0.53 | 0.062 |
| HB(million/µL) | 1.02 | 0.01 | 1.02 | 0.01 | 0.97 | 0.019 | - | - |
| BUN (million/µL) | 1.0 | 0.04 | 1.00 | 0.005 | 1.00 | 0.005 | - | - |
| Creatinine (mg/dL) | - | - | - | - | 0.89 | 0.001 | - | - |
| NA(mmol/L) | 0.99 | 0.001 | - | - | 0.99 | 0.001 | - | - |
| K(mmol/L) | 1.00 | 0.05 | - | - | 1.01 | 0.08 | - | - |
| Albumin(g/dL) | 0.94 | 0.049 | 0.93 | 0.05 | 0.93 | 0.15 | 0.93 | 0.05 |

- Not selected as important factor

Death due to Pulmonary Embolism is the interested event, Death due to Sepsis is the competing event
